# Assessing laboratory specimen losses for the city of Johannesburg, South Africa

**DOI:** 10.4102/phcfm.v17i1.4907

**Published:** 2025-07-30

**Authors:** Naseem Cassim, Ernest P. Buthelezi, Somayya Sarang, Sadhaseevan Moodly, Lucia Hans, Lindi-Marie Coetzee

**Affiliations:** 1 Wits Diagnostic Innovation Hub (DIH), Faculty of Health Sciences, University of the Witwatersrand, Johannesburg, South Africa; 2 National Priority Programme, National Health Laboratory Service, Johannesburg, South Africa; 3 Gauteng Department of Health (GDoH), Johannesburg, South Africa; 4 National Health Laboratory Service, Johannesburg, South Africa; 5 Department of Molecular Medicine and Haematology, Faculty of Health Sciences, University of the Witwatersrand, Johannesburg, South Africa

**Keywords:** pathology value chain, losses, laboratory information system, rejection, repository, primary health care

## Abstract

**Background:**

Specimen losses across the pathology value chain (PVC) result in missed diagnostic opportunities. It is difficult to fully assess these due to the current paper-based systems, with tracking of specimens only possible on the laboratory information system (LIS).

**Aim:**

This study aimed to assess specimen losses using the paper-based register.

**Setting:**

Randomly selected Primary health care (PHC) facilities, City of Johannesburg, South Africa.

**Methods:**

The retrospective descriptive study design was used to scan 1,000 barcodes from facilities in sub-districts A to G. Data was limited to barcodes from the request form and excluded surveillance testing. Matching data from the laboratory repository was extracted. PVC losses were assessed by determining the percentage of scanned barcodes that had a registered, tested, reviewed and/or rejected date. The analysis was stratified according to sub-district, health facility type and test code.

**Results:**

The dataset analysed included 33 867 barcodes with 121 697 test codes, equating to 3.59 tests per barcode. Matching registered, tested and reviewed dates were detected for 33 107 (97.76%) barcodes. In total, a rejection for one or more test codes was detected for 1,961 barcodes (5.79%). At the sub-district level, between 95.95% (D) and 98.90% (E) of barcodes were reviewed. The rejection rate ranged from 3.27% (F) to 10.93% (D). For community health centres and clinics, 97.37% and 97.97% of the barcodes had a matching reviewed date.

**Conclusion:**

PVC losses reported were 4.05%, excluding rejections (5.79%), with slightly higher levels noted at the sub-district level.

**Contribution:**

The continuous audit of PVC losses is recommended.

## Introduction

Laboratories play a significant role in the diagnosis, monitoring and treatment of diseases.^1^ Multiple factors affect specimen losses in the pathology value chain (PVC), such as the capacity of the laboratory workforce, laboratory infrastructure, availability of equipment and specimen collection materials and pre- and post-analytical factors.^1,2^ The total testing process (TTP) in the PVC comprises three phases: (1) pre-analytical, (2) analytical, and (3) post-analytical.^3,4^ The pre-analytical phase includes all procedures performed before the specimen reaches the laboratory, that is specimen collection, packaging, storage and transport.^3,4^ In contrast, the analytical phase refers to the actual laboratory test analysis (sample preparation, testing and troubleshooting).^3,4^ The post-analytical phase includes test result review and release to the health care worker (HCW).^3,4^ It has been estimated that most errors are because of pre-analytical factors (46% – 68.2%) followed by post-analytical factors (18.5% – 47%).^2^ Errors in the analytical phase, which include sample preparation errors, equipment malfunction and instrument downtime, have been significantly reduced because of state-of-the-art technology (closed, fully automated systems) and a focus on quality through both internal and external quality control measures.^2,5^

Losses in the PVC are defined as specimens collected from patients attending a health facility for laboratory testing that have been registered in the paper-based register but are not found in the laboratory information system (LIS) or that have been subsequently rejected.^2,5,6,7^ This is confirmed by the primary health care (PHC) laboratory handbook that states that specimens should be collected, packaged, stored and prepared for transportation, and the laboratory results should be received.^6^ Where this has not taken place, it can be defined as a PVC loss.^6^ This implies that specimens were collected for which no clinical decision could be made for patient management. These PVC specimen losses may occur at multiple points of the TTP and result in missed diagnostic opportunities (MDO) that potentially affect patient clinical management.^8,9^ In addition, PVC losses also result in the patient having to return to the PHC facility for an additional visit for a repeat specimen to be collected. A local study indicated that patients accessing antiretroviral therapy (ART) incurred expenses that consisted of transportation as well as payment for medical care.^10^ Therefore, PVC losses should be minimised to reduce MDO and retain patients in care.

The reasons for specimen losses in the PVC may include: incomplete request forms (such as the failure to provide mandatory data variables), specimen collection errors, specimens lost in transit, data capturing errors, haemolysed or clotted specimens, as well as analytical challenges.^4,11^ There are many pre-analytical challenges between the health facility and the laboratory that cannot be assessed because of the paper-based recording systems and the absence of specimen tracking outside the LIS.^4,11^ The paper-based systems make it difficult to assess PVC losses outside of electronic data generated by the laboratory.

The LIS belongs to the class of application software intended for the storage and management of information obtained in the course of the work of the laboratory service.^12^ The systems are used to control and manage specimens, standards, test results, reports, laboratory staff and instruments, as well as workflow automation.^12^ Within the LIS, electronic dates and times are allocated when specimens are registered, tested and reviewed. In addition, when specimens are referred within the national laboratory network, electronic dates and times are allocated when the specimens are referred and received at the testing laboratory. Furthermore, when a test is rejected, the date of rejection is also captured on the LIS. The electronic date/time information is vital in the laboratory network to assess turnaround time performance.^13,14^ Furthermore, the outstanding testing can be identified using the LIS.

There have been platforms such as eLABS that are used to track specimens across the PVC.^15^ eLABS is a mobile workflow solution where HCWs are able to request tests and capture a sample barcode as well as the patient details.^15,16^ The specimens are tracked from acquisition by the HCW and at each step of the laboratory workflow.^15,16^

Once results are available, HCWs are notified on the mobile application.^15,16^ Through use of ‘Results for Action’, facilities become aware of results that require triaging quicker than traditional methods of result delivery.^15,16^ In addition, the eLABS patient support module (PSM) sends test results, relevant educational messages and appointment reminders directly to patients.^15,16^ However, these systems have only been implemented for selected tests and facilities in health districts supported by both the Centers for Disease Control and Prevention (CDC) and the President’s Emergency Plan for AIDS Relief (PEPFAR).^15,17^ There are also initiatives to develop a Health Patient Registration System (HPRS) in South Africa for use by PHC facilities.^6^ Despite the existence of these systems, the clinic-laboratory interface (CLI) is still paper based.^18,19^ Order entry is an application that enables HCWs to create electronic orders within the facility. This will replace traditional pen-and-paper request forms and registers for ordering laboratory investigations.^20,21,22^ With order entry, the HCW can select the tests required, with the patient and HCW information populated by a system such as the HPRS.^21^ The order is electronically transmitted to the LIS and the patient result outcomes are returned electronically – removing the need for paper-based systems.^21,23^ The absence of order entry systems in a South African health setting makes it difficult to objectively assess PVC specimen losses as data are only captured electronically once registered in the LIS.

Assessing PVC specimen losses using only LIS data may be an underestimate as it does not account for losses prior to registration at the local laboratory. Therefore, the facility register that records the request form barcode for every patient offers an objective mechanism to assess PVC specimen losses outside the LIS. An objective audit of PVC losses is needed for all tests included as part of the PHC package of services in a local setting, given the limited data available.^24^

## Research methods and design

### Study design

A retrospective descriptive study design was used. This study aimed to assess specimen losses in the PVC using barcodes scanned from the PHC facility registers and matched laboratory repository data for the City of Johannesburg (COJ), South Africa at two endpoints: (1) registered/tested/reviewed and (2) rejected. The data sources included the scanned barcodes and an extract from the Corporate Data Warehouse (CDW) of the National Health Laboratory Service (NHLS). This data warehouse serves as a centralised repository for all laboratory data collected by the NHLS from public sector health facilities across South Africa. The CDW ingests laboratory data and uses data warehousing and integration tools to conform data for monitoring purposes. Furthermore, the CDW provides access to laboratory records for approximately 80% of South Africa’s population, facilitating informed decision-making in disease surveillance, healthcare interventions and policy development.

### Setting

The study was conducted using PHC facilities in the COJ, Gauteng province, South Africa.^25^ In 2021, the COJ had an estimated 5.87 million people, making it the biggest metropolitan municipality by population size in South Africa.^26^ The PHC laboratory handbook provides a step-by-step guide for facilities regarding the correct completion of request forms and compliance with the minimum clinical data set requirements.^6^ Based on clinical assessment of the patient, HCWs need to identify the appropriate and relevant laboratory tests to be performed using evidence based clinical guidelines.^6,24^ When ordering laboratory tests, HCWs are required to complete either the N1 PHC or the N2 Cytology laboratory request form for all specimens submitted to a NHLS laboratory.^6^ The following mandatory information is required: (1) patient’s folder or HPRS number, (2) patient’s national identity (ID) number or passport number, (3) patient’s name, (4) patient’s surname, (5) patient’s date of birth, (6) patient’s gender, (7) HCW’s name, (8) HCW’s Health Professions Council of South Africa (HPCSA) or South African Nursing Council (SANC) number, (9) HCW’s signature, (10) collection date, (11) tests requested, and (12) concise description of the clinical problem and/or diagnosis.^6^ Each request form has a unique barcode. All facilities utilising NHLS services use the N1 PHC and N2 Cytology laboratory request forms that each contain a unique barcode. The N1 form barcode is 10 characters in length, with four letters followed by four numbers and ending with the ‘P’ prefix for example, ABCD1000P. The prefix indicates that requests were made by PHC facilities.

Prior to transport to the local laboratory, all specimens should be recorded in the facility specimen register.^6^ This includes placing a barcode sticker from the request form in the facility register (N4).^6^ The following information is also captured in the register: (1) date and time, (2) patient folder/(HPRS) number, and (3) ticking the appropriate disciplines for the test(s) requested (refer to N1/N2 request form). This entire process is paper based, with information captured in the register by the HCW as specimens are collected and prepared for collection by the courier. Data are only electronically captured (on the LIS) upon arrival at the receiving laboratory. The specimens that were scanned are served by a range of laboratories that are based at district, regional and tertiary hospitals with the COJ. The testing was not provided at a single laboratory.

### Study population

Facilities from the seven sub-districts were selected for the study: (1) Diepsloot or Midrand, (2) Randburg, (3) Roodepoort, (4) Greater Soweto, (5) Sandton or Alexandra, (6) Inner city and (7) Eldorado Park, Ennerdale or Orange farm.^25^ There are 117 PHC facilities in the COJ, of which 13, 10, 13, 29, 11, 14 and 27 are situated in sub-districts A to G, respectively, according to the district health information system (DHIS) organisational list.^27^

### Sample size

Overall, there are 102 clinics and 15 community health centres (CHC) in this area.^27^ The sample frame was calculated using 117 health facilities. To achieve a 95% confidence interval (CI), the sample size required was calculated as 383 barcodes. All testing from these health facilities was performed by the NHLS, which is mandated to provide diagnostic services to the national, provincial and local departments of health through its country-wide network of quality assured diagnostic laboratories.^28^ There are 27 laboratories in Gauteng province.^28^

### Sampling

Stratified random sampling was used to select PHC facilities from each sub-district. Given the limited study funding available, the aim was to conduct barcode scanning for at least three facilities per sub-district. The DHIS organisational list was used conduct the random sampling for each sub-district.^29^ The Microsoft Excel (Microsoft Corporation, Redmond, CA, USA) random number generator was used.

### Inclusion criteria

From each facility, 1000 barcodes were scanned, with only barcodes in the format of the N1 request form requested between March 2023 and May 2024 eligible for analysis. Tests performed for surveillance by the National Institute of Communicable Diseases (NICD) were excluded (identified by a test code with a ‘T’ prefix).

### Data collection

The barcodes were scanned using a hand-held scanner that used a trigger mechanism to facilitate electronic capturing from paper-based records. The requests included various specimen types and test codes. This study was conducted separately from patient care as the paper-based results were delivered to the health facility independently of this study. The scanning was performed by Gauteng Department of Health staff after approval had been obtained from the facility manager and the NHRD. The scanned barcodes were collated into a single Microsoft Excel worksheet (Microsoft Corporation, Redmond, CA, USA).

### Data preparation

For the scanned barcodes, matching data were requested from the CDW to assess whether specimens had a corresponding LIS date and time recorded. The CDW data extract included the following variables: (1) barcode number, (2) test code, (3) test code description, (4) registered date, (5) tested date, (6) reviewed date, and (7) rejected date (only if the specimen was rejected).

### Data analysis

The data extracted from the CDW was combined with the corresponding scanned barcodes using the Microsoft Access Microsoft Corporation (Redmond, WA, USA) relational database with a right outer join. This analysis was conducted using Statistical Analysis Software (SAS) 9.4 (Cary, NC, USA). For each date variable, data were coded as a binary variable (1: value provided or 0: no matching values). The rejection date was used to quantify how many barcodes were rejected and was reported as the rejection rate (RR). The registered, tested and reviewed dates were used to determine what percentage of barcodes were captured on the LIS, with missing values assumed to constitute PVC losses. The percentage losses in the PVC were further stratified by sub-district and health facility type. The 20 most frequently recorded LIS test codes were also analysed, with the remainder categorised as ‘Other’. The LIS test codes are based on the code table rules defined during LIS configuration, which may differ from how tests are requested on the N1 request form. Pathology value chain losses for the study consisted of both specimens that were captured in the facility register but not found in the laboratory repository as well as specimen that were rejected on the LIS.

### Ethical considerations

Ethical clearance to conduct this study was obtained from the University of the Witwatersrand (No. M240328). Permission to conduct the study was also received from the Gauteng Province Health Research Committee (No. GP_202404_092).

## Results

**FIGURE 1:**
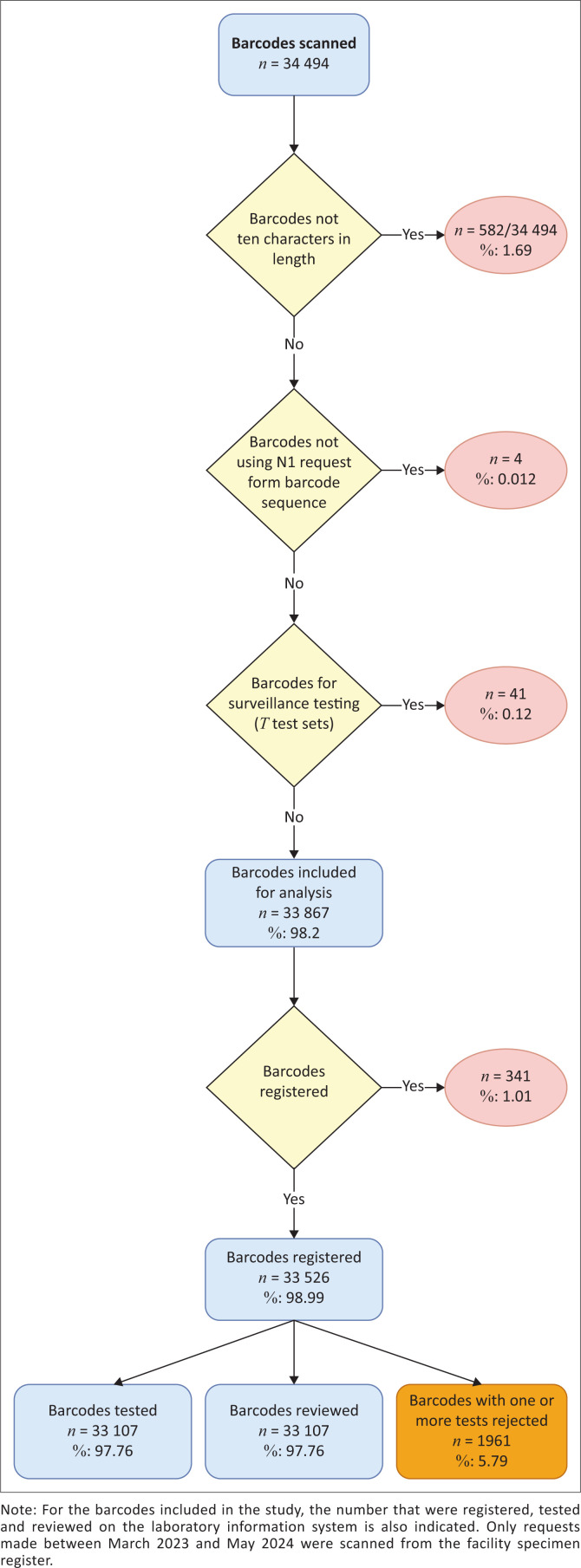
Decision tree depicting the barcodes scanned from randomly selected Primary Health Care facilities in the City of Johannesburg as well as the reasons for exclusion.

In all, 34 PHC facilities were selected for the study from 117 PHC facilities in Gauteng province (Table 1). These included: 5 (Bophelong Ivory Park, Diepsloot South clinic, Ebony Park CHC, Hikhensile and OR Tambo CHC), 5 (Bosmont clinic, Randburg clinic, Riverlea Major clinic, Sophiatown clinic and Westbury CHC), 4 (Davidonsville clinic, Discoverers CHC, Florida clinic and Rex Street Clinic), 3 (Chiawelo CHC, Lillian Ngoyi CHC and Mofolo CHC), 5 (Alexandria 8th Avenue clinic, Alexandria CHC, East bank clinic, Orchards clinic and Thokomngoma clinic), 5 (Esselen clinic, Hillbrow CHC, Jeppe clinic, Kibler park clinic and Yeoville clinic) and 7 (Cosmo clinic, Ennerdale Extension 8, Freedom Park clinic, Lenasia Extension 13, Lenasia South Civic Centre, Stretford CHC and Thulamntwana) facilities for sub-districts A to G, respectively. There were 34 494 barcodes scanned for this study. Of these, 582/34 494 (1.69%) were excluded because of barcode length. Four barcodes (4/34 494) were excluded as the ‘P’ suffix was missing (0.01%). Test codes associated with surveillance were also excluded (*n* = 41/34 494; 0.12%) (Figure 1). An example of surveillance testing would include measles testing during an outbreak. The final study dataset analysed included 33 867/34 494 (98.18%) barcodes that had 121 697 associated test codes. On average, there were 3.59 test codes per barcode. Matching registered dates from the laboratory repository were detected for 33 526/33 867 (98.99%) barcodes. Of these, a tested and reviewed date was not found for 419/33 526 barcodes (1.24%). A rejection for one or more test codes was detected for 1961/33 867 barcodes (5.79%).

**TABLE 1:** Analysis of barcodes and test codes per sub-district that were registered, tested and reviewed.

Sub-district	Barcodes		Barcodes registered, tested and reviewed		Barcodes with one or more rejections		Test codes		Test codes per barcode
	*n*	%	*n*	%	*n*	%	*n*	%	
A	5307	15.67	5213	98.23	196	3.69	15 130	12.43	2.85
B	4885	14.42	4775	97.75	351	7.19	16 763	13.77	3.43
C	3992	11.79	3913	98.02	212	5.31	11 783	9.68	2.95
D	2991	8.83	2870	95.95	327	10.93	16 521	13.58	5.52
E	5009	14.79	4954	98.90	270	5.39	18 052	14.83	3.60
F	5014	14.80	4929	98.30	164	3.27	17 743	14.58	3.54
G	6669	19.69	6453	96.76	441	6.1	25 705	21.112	3.85

**Total**	**33 867**	**100.00**	**33 107**	**97.76**	**1961**	**5.79**	**121 697**	**100.00**	**3.59**

Note: The number of rejections was also assessed (one or more test codes rejected per barcode). Data are reported for barcodes scanned at randomly selected primary health care facilities in the City of Johannesburg, South Africa, across sub-districts A to G between March 2023 and May 2024 from the Facility Specimen Register.

A, Diepsloot/Midrand; B, Northcliff/Randburg; C, Roodepoort; D, Greater Soweto; E, Sandton/Alexandra; F, Inner City; G, Ennerdale/Orange Farm.

### Sub-district analysis

The contribution of barcodes per sub-district ranged from 8.83% (D: *n* = 2991/33 867) to 19.69% (G: *n* = 6669/33 867) (Table 1). The percentage of barcodes that were registered, tested and reviewed ranged from 95.95% (D) to 98.90% (E), with an average of 97.76%. The contribution of test codes per sub-district ranged from 9.68% (C) to 21.12% (G). The number of test codes per barcode varied from 2.85 (A) to 5.52 (D). For barcodes with a reviewed date, the RR ranged from 3.27% (F) to 10.93% (D).

### Facility type analysis

Overall, 35.53% of barcodes originated from CHC (Table 2). For CHC, 97.37% of barcodes were registered, tested and reviewed compared to 97.97% for clinics. The contribution of test codes by CHC versus clinics was 41.79% versus 58.21%. On average, 4.23 test codes were requested per barcode for CHC with 3.24 reported for clinics. The RR was 7.20% for CHC compared to 5.02% for clinics.

**TABLE 2:** Analysis of barcodes per facility type that were registered, tested and reviewed.

Facility type	Barcodes		Barcodes registered, tested and reviewed		Barcodes with one or more rejections		Test codes		Test codes per barcode
	*n*	%	*n*	%	*n*	%	*n*	%	
Community health centres	12 034	35.53	11 717	97.37	866	7.20	50 863	41.79	4.22
Clinics	21 833	64.47	21 390	97.97	1095	5.02	70 834	58.21	3.24
**Total**	**33 867**	**100.00**	**33 107**	**97.76**	**1961**	**5.79**	**121 697**	**100.00**	**3.59**

Note: The number of rejections was also assessed (for one or more test codes per barcode). Data are reported for barcodes scanned at randomly selected primary health care (PHC) facilities in the City of Johannesburg, South Africa, between March 2023 and May 2024 from the Facility Specimen Register.

### Test code analysis

The top 20 test codes collectively contributed 105 729/121 697 (86.88%) of the total, with the remaining test codes categorised as ‘Other’ (13.12%) (Table 3). The most requested test codes corresponded to creatinine (19.26%), followed by human immunodeficiency virus (HIV) viral load (13.53%), serum indices (12.00%), total cholesterol (6.42%) and treponema pallidum antibodies (5.07%).

**TABLE 3:** Analysis of test codes reported with the percentage contribution indicated.

No.	Test group	Test codes	
		*n*	%
1	Creatinine	23 444	19.26
2	HIV viral load	16 462	13.53
3	Other	15 968	13.12
4	Serum indices	14 604	12.00
5	Total cholesterol	7810	6.42
6	*Treponema pallidum* antibodies	6173	5.07
7	Haemoglobin	4636	3.81
8	CD4	4629	3.80
9	Prostate-specific antigen	3986	3.28
10	GeneXpert Ultra	3681	3.02
11	Hepatitis B surface antigen	3457	2.84
12	Potassium	3318	2.73
13	HbA1c	2536	2.08
14	Sodium	2373	1.95
15	Full blood count	1741	1.43
16	Urea	1328	1.09
17	Chloride	1183	0.97
18	Bicarbonate	1181	0.97
19	Triglycerides	1099	0.90
20	LDL	1060	0.87
21	Thyroid-stimulating hormone	1028	0.84

**Total**		**121 697**	**100.00**

Note: The top 20 test codes based on volumes are reported with the remainder categorised as Other. Data are reported for barcodes scanned at randomly selected primary health care facilities in the City of Johannesburg, South Africa, for sub-districts A to G between March 2023 and May 2024 from the Facility Specimen Register.

HIV, human immunodeficiency virus; HbA1c, glycated haemoglobin; LDL, low-density lipoprotein.

## Discussion

This is one of the first studies to assess losses in the PVC for a large sample size. Most of the studies that have analysed losses in the PVC outside South Africa have been described for surgical specimens.^7,30,31^ The study findings revealed that PVC losses where barcodes were scanned in the facility register but not found in the laboratory repository was 1.01%. However, there was a further 1.24% that did not have a corresponding tested/reviewed data. In addition, 5.79% of barcodes had one or more test codes that were rejected. At the sub-district level, barcodes without a corresponding tested/reviewed date increased to 4.11%. Similarly, the RR increased to 10.9% when analysed at the sub-district level. Carmack et al. conducted a systematic review, which highlighted the limited research in this area, with an average of two articles per year discussing lost specimens.^31^ It is, therefore, difficult to interpret PVC losses for this study comparatively.

It has been reported that the RR for CD4 testing (excluding electronic gatekeeping) ranged from 2.2% to 4.4% at the provincial level in 2019.^32^ Similarly, a local study at an academic hospital reported that for HIV 1/2 serology, the RR was as high as 11.1%.^33^ In contrast, an analysis of rejections at the Green Point complex tuberculosis laboratory reported a rate of 2.3% over a 2-year period.^34^ The National Priority Programme (NPP) internal report for April 2024 reported a RR of 4.5%, 4.7%, 2.7% and 2.7% for Xpert MTB/RIF, CD4, HIV viral load and HIV DNA PCR testing, respectively.^35^ The RR is test dependent and our findings are within the range reported locally.^32,33,34,35^ However, at the sub-district level, higher RR was reported for Region D, which warrants further investigation. Clearly, the interpretation of RR is context and test specific, with a host of other factors that may play a role.

For this study, losses in the PVC were similar for clinics and CHC. Overall, losses were slightly higher for CHC as well as reporting a higher rate of test codes per barcode. This finding is expected as CHC have a broader package of services that includes a 24-h midwife obstetric unit (MOU), emergency unit and the provision of health support services (physical rehabilitation by physical therapist and occupational therapist, speech and hearing therapy, dietetics, social worker support) as well as oral health services.^36^ The higher RR for CHC could also be linked to the broader package of services as well as a larger health facility servicing a broader catchment area.^36^

According to the PHC essential laboratory list (ELL) and updated 2020 Standard Treatment Guidelines (STG) and Essential Medicines List (EML), all top 20 test codes reported for the study are compliant with testing recommendations for this level of care.^6,24^ Some testing such as Serum Indices were not indicated in the PHC ELL, but were included in the standard of care in the 2020 STG & EML.^24^ This is consistent with findings by Mahommed et al. that reported that between 98.2% and 99.1% of testing in the COJ was ELL compliant.^37^ This highlights the need for PHC ELL and laboratory handbooks to be updated to ensure consistency with updated guidelines.^24,37,38^ For example, severe acute respiratory syndrome coronavirus 2 (SARS-CoV-2) testing should have been included during the coronavirus disease 2019 (COVID-19) pandemic.^39,40^ The PHC ELL should become a real-time document that is continually updated based on new outbreaks as well as guideline changes.^24,38^ South Africa should follow the example set by Nigeria that is in line with the World Health Organization (WHO) recommendation for the development and implementation of the National Essential Diagnostics List (NEDL) to facilitate the availability of diagnostic services.^41,42^

There are multiple reasons why specimens collected at the health facility are not recorded in the laboratory repository. It has been reported that most errors occur during the pre-analytical and post-analytical phases of the CLI.^2,43^ Some of these pre-analytical factors contributing specifically to barcodes not being recorded in the LIS could include: barcodes captured in the register without being collected, specimens collected but not given to the courier or specimens lost during courier transport or at the receiving office prior to capture in the LIS. Unfortunately, this study did not attempt to provide clarity on the exact reason for PVC specimen losses, given the absence of a specimen tracking system. As this was a retrospective study, it would be too late to conduct a vertical audit for the barcodes that were registered but not tested or reviewed.

Continuous audits are warranted, given the wider range in PVC losses, which was not statistically significant at the sub-district level, in particular. The routine and continuous audit of PVC losses would facilitate performance of root cause analysis by both the Department of Health and diagnostic service providers. After the investigation, corrective actions should be taken to reduce losses that will ultimately result in both improved patient care and reduced wastage. This could be conducted routinely by using paper-based facility registers to scan barcodes. To avoid the manual analysis of PVC losses, it is proposed that a cloud-based solution be developed by the CDW. Scanned barcode lists could be uploaded on a portal, processed via an extract, transformed and loaded (ETL) in a data warehouse, compared to LIS data, and used to generate PVC loss reports and dashboards for electronic access or email distribution. These dashboards could also be included as part of the overall quality management system. This would make it possible for managers to conduct a more automated audit of PVC losses. The only investment required would be for laboratory coordinators to be provided with handheld barcode scanners and some development within the CDW. This study could also be further enhanced should order entry be implemented, as routine monitoring of PVC losses would be facilitated.^21,44,45^ Order entry would potentially extend the capacity to allow for financial reconciliation and better tracking of specimens. This study also demonstrated the value of well-curated laboratory data.^46^

### Limitations

A limitation of this study is that the PVC losses reported for health facilities in the COJ may not be generalisable to other settings. The process is also reliant on the capture of barcodes in the facility register using a handheld scanner, which is necessary to reduce the possibility of transcription errors. The urban study setting may not be indicative of PVC losses in more rural areas. Another challenge is that there may be some health facilities in other provinces, where barcodes are not routinely used. It is difficult to assess the reasons why some scanned barcodes were not found in the laboratory repository. Without an audit at the time of testing, it is difficult to determine the exact cause of PVC losses. The data captured for rejections were reported on the LIS; however, the data extract provided for the study did not include the reason. It is recommended that a follow-up study be conducted that assesses the specific reason for rejections that could guide corrective action such as training, changes to the handbook and improved CLI communication. Additional analysis of data at the PHC level could reveal whether pre-analytical factors contribute to the overall RR.^2,5^ It has been reported that for CD4 and HIV viral load testing, the most common rejection reasons were electronic gatekeeping and unsuitable specimens, according to the NPP internal report.^35^ It would, however, be difficult to generalise these findings to broader PHC testing.

## Conclusion

This study demonstrated an objective methodology independent of the LIS, showing that 4.05% of specimens not tested/reviewed, with a further 5.79% with a rejection. These findings indicate the need for implementation of continuous or frequent/periodic audits of PVC specimen losses in the public health sector. It would be possible to use the existing facility registers to routinely scan barcodes to conduct an audit of PVC losses. Automated solutions could be developed to enable audits to be conducted more autonomously and to detect higher levels of PVC losses earlier. This in turn would allow for more rapid and targeted interventions.

## References

[1] Tuijn CJ, Msoka E, Mushi DL, Sumari-de Boer M, Chilongola J, Van den Broek A. The interface between clinicians and laboratory staff: A field study in northern Tanzania. Afr J Lab Med. 2014;3(1):126. 10.4102/ajlm.v3i1.126 29043178 PMC5637763

[2] Plebani M. Errors in clinical laboratories or errors in laboratory medicine? Clin Chem Lab Med. 2006;44(6):750–759. 10.1515/cclm.2006.123 16729864

[3] Sharaki O, Abouzeid A, Hossam N, Elsherif Y. Self-assessment of pre, intra and post analytical errors of urine analysis in Clinical Chemistry Laboratory of Alexandria Main University Hospital. Saudi J Health Sci. 2014;3(2):96–102. 10.4103/2278-0521.134863

[4] Plebani M, Sciacovelli L, Aita A. Quality indicators for the total testing process. Clin Lab Med. 2017;37(1):187–205. 10.1016/j.cll.2016.09.015 28153366

[5] Plebani M. The detection and prevention of errors in laboratory medicine. Ann Clin Biochem. 2010;47(2):101–110. 10.1258/acb.2009.009222 19952034

[6] National Department of Health (NDOH), National Health Laboratory Service (NHLS). Primary healthcare laboratory handbook: A step-by-step guide [homepage on the Internet]. 2018 [cited 2024 Feb 12]. Available from: https://www.idealhealthfacility.org.za/App/Document/Download/20

[7] Sandbank S, Klein D, Westreich M, Shalom A. The loss of pathological specimens: Incidence and causes. Dermatol Surg. 2010;36(7):1084–1086. 10.1111/j.1524-4725.2010.01587.x 20533941

[8] Goyder CR, Jones CH, Heneghan CJ, Thompson MJ. Missed opportunities for diagnosis: Lessons learned from diagnostic errors in primary care. Br J Gen Pract. 2015;65(641):e838–e844. 10.3399/bjgp15X687889 26622037 PMC4655738

[9] Haeri Mazanderani A, Moyo F, Sherman GG. Missed diagnostic opportunities within South Africa’s early infant diagnosis program, 2010–2015. PLoS One. 2017;12(5):e0177173. 10.1371/journal.pone.0177173 28493908 PMC5426641

[10] Rosen S, Ketlhapile M, Sanne I, DeSilva MB. Cost to patients of obtaining treatment for HIV/AIDS in South Africa. S Afr Med J. 2007;97(7):524–529. 17805455

[11] Hawkins R. Managing the pre-and post-analytical phases of the total testing process. Ann Lab Med. 2012;32(1):5–16. 10.3343/alm.2012.32.1.5 22259773 PMC3255486

[12] Skobelev D, Zaytseva T, Kozlov A, Perepelitsa V, Makarova A. Laboratory information management systems in the work of the analytic laboratory. Meas Tech. 2011;53(10):1182–1189. 10.1007/s11018-011-9638-7

[13] Cassim N, Tepper ME, Coetzee LM, Glencross DK. Timely delivery of laboratory efficiency information, Part I: Developing an interactive turnaround time dashboard at a high-volume laboratory. Afr J Lab Med. 2020;9(2):1–9. 10.4102/ajlm.v9i2.947 PMC7203318 32391244

[14] Coetzee LM, Tepper ME, Perelson L, Glencross DK, Cassim N. Timely delivery of laboratory efficiency information, Part II: Assessing the impact of a turn-around time dashboard at a high-volume laboratory. Afr J Lab Med. 2020;9(2):1–8. 10.4102/ajlm.v9i2.948 PMC7203269 32391245

[15] Chigudu K, Isherwood L, Marange F, Gorogodo B, Sofute N, Kadira B. eLABS: Digital health intervention strengthens the clinical-laboratory interface for the HIV viral load value chain in the Luanshya district. In: 4th International African Society for Laboratory Medicine (ASLM 2018). Abuja; 2018 [cited 2025 Jan 25]. Available from: https://www.researchgate.net/publication/350884496_eLABS_Digital_Health_Intervention_Strengthens_the_Clinical-Laboratory_Interface_for_the_HIV_Viral_Load_Value_Chain_in_the_Luanshya_District_Zambia_Background_Results_Conclusions_Methods_Acknowledgemen

[16] Mezzanine. eLABS: Faster lab result turnaround time, better patient care [homepage on the Internet]. 2025 [cited 2025 Mar 27]. Available from: https://mezzanineware.com/digital-productivity-technology/healthcare-technology-solutions/laboratory-improvement-technology/

[17] Cassim N, Olsen F, Stewart-Isherwood L, Da Silva MP, Stevens WS. Assessing the cost and utilization of SMS printers by primary health care facilities: Lessons learned from South Africa. J Public Health Africa. 2023;14(4):2253. 10.4081/jphia.2023.2253 PMC10280246 37347071

[18] Tucker TJ, Manyike PT. Improving the clinic-laboratory-interface in the context of HIV diagnosis, treatment, and monitoring. Curr Opin HIV AIDS. 2017;12(2):105–111. 10.1097/COH.0000000000000350 28079593

[19] National Department of Health (NDOH). Integrated Clinical Services Management (ICSM) [homepage on the Internet]. 2023 [cited 2024 Feb 12]. Available from: https://knowledgehub.health.gov.za/system/files/elibdownloads/2023-04/Integrated%252520Clinical%252520Services%252520Management%252520%252520Manual%2525205th%252520June%252520FINAL.pdf

[20] Dixon BE, Zafar A. Inpatient Computerized Provider Order Entry (CPOE) – Findings from the AHRQ Health IT Portfolio. 2009 [cited 2025 Jan 25]. Available from: https://scholarworks.indianapolis.iu.edu/server/api/core/bitstreams/7b0a8f04-bff3-45d4-8d8e-374febcb2b67/content

[21] Aarts J, Ash J, Berg M. Extending the understanding of computerized physician order entry: Implications for professional collaboration, workflow and quality of care. Int J Med Inform. 2007;76:S4–S13. 10.1016/j.ijmedinf.2006.05.009 16798068

[22] Cassim N, Ramdin N, Moodly S, Glencross DK. Cost of running a full-service receiving office at a centralised testing laboratory in South Africa. Afr J Lab Med. 2022;11(1):1–8. 10.4102/ajlm.v11i1.1504 PMC9350462 35937761

[23] Teich JM, Hurley JF, Beckley RF, Aranow M, editors. Design of an easy-to-use physician order entry system with support for nursing and ancillary departments. In: Proceedings of the Annual Symposium on Computer Application in Medical Care. American Medical Informatics Association; 1992 [cited 2025 Jan 25]. Available from: https://pubmed.ncbi.nlm.nih.gov/1483016/PMC2248034 1483016

[24] National Department of Health (NDOH). Standard treatment guidelines and essential medicines list for South Africa [homepage on the Internet]. 2020 [cited 2024 Feb 12]. Available from: https://knowledgehub.health.gov.za/system/files/elibdownloads/2023-09/Primary_20Healthcare_20STGs_20and_20EML_207th_20edition_20-_202020-v3.0.pdf

[25] Abrahams C, Everatt D. City profile: Johannesburg, South Africa. Environ Urban ASIA. 2019;10(2):255–270. 10.1177/0975425319859123

[26] City of Joburg. City of Johannesburg integrated development plan 2021-26 [homepage on the Internet]. 2021 [cited 2025 Mar 27]. Available from: https://joburg.org.za/documents_/Documents/2021-2026%20Final%20IDP/2021-26%20FINAL%20IDP%2021May%202021.pdf

[27] Williamson L, Stoops N, Heywood A. Developing a District Health Information System in South Africa: A social process or technical solution? MEDINFO 2001. London: IOS Press, 2001; p. 773–777. 11604842

[28] National Health Laboratory Service (NHLS). Annual report 2022/23 [homepage on the Internet]. Johannesburg: National Health Laboratory Service (NHLS); 2023 [cited 2025 Jan 24]. Available from: https://www.nhls.ac.za/wp-content/uploads/2023/11/241023-NHLS-Annual_Report.pdf

[29] Garrib A, Stoops N, McKenzie A, et al. An evaluation of the district health information system in rural South Africa. S Afr Med J. 2008;98(7):549–552. 18785397

[30] Nakhleh RE. Lost, mislabeled, and unsuitable surgical pathology specimens. Pathol Case Rev. 2003;8(3):98–102. 10.1097/01.PCR.0000065693.59517.7E

[31] Carmack HJ, Lazenby BS, Wilson KJ, Bakkum-Gamez JN, Carranza L. Lost, mislabeled, and mishandled surgical and clinical pathology specimens: A systematic review of published literature. Am J Clin Pathol. 2024; 162(4):349–355. 10.1093/ajcp/aqae055 38738289

[32] Cassim N, Buthelezi EP, Coetzee LM, Glencross DK. Assessing CD4 rejections across a national laboratory service for 2018 in South Africa: Highlighting the importance of adherence to national handbook guidelines. J Public Health Afr. 2022;13(1):1278. 10.4081/jphia.2022.1278 35720799 PMC9202455

[33] Reddy B, Cassim N, Treurnicht F, Makatini Z. Factors influencing the high rejection rates of HIV 1/2 serology samples at Charlotte Maxeke Johannesburg Academic Hospital and the cost implications. S Afr J HIV Med. 2022;23(1):1326. 10.4102/sajhivmed.v23i1.1326 PMC8832030 35169497

[34] Zerbini S, Singh S, Botha M, Ghebrekristos Y, Opperman CJ. Specimen rejection in a high-throughput TB laboratory: A descriptive study. S Afr Med J. 2023;113(10):6–7. 10.7196/SAMJ.2023.v113i10.1364 37881905

[35] National Health Laboratory Service (NHLS). National Priority Programs April 2024 Operational Committee Report. Johannesburg: National Health Laboratory Service (NHLS); 2024.

[36] National Department of Health (NDOH). Ideal clinic and CHC framework [homepage on the Internet]. 2024 [cited 2024 Feb 12]. Available from: https://www.idealhealthfacility.org.za/App/Document/Download/386

[37] Mahomed O, Cassim N. Appropriateness of laboratory expenditure for primary health care facilities across South Africa. Afr J Prim Health Care Fam Med. 2023;15(1):3740. 10.4102/phcfm.v15i1.3740 37403680 PMC10319931

[38] National Department of Health (NDOH). ART clinical guidelines for the management of HIV in adults, pregnancy and breastfeeding, adolescents, children, infants and neonates [homepage on the Internet]. Pretoria: National Department of Health (NDOH); 2023 [cited 2024 Jan 18]. Available from: https://knowledgehub.health.gov.za/system/files/elibdownloads/2023-07/National%20ART%20Clinical%20Guideline%20AR%204.5%2020230713%20Version%204%20WEB.pdf

[39] National Institute for Communicable Diseases (NICD). First case of Covid-19 announced – An update [homepage on the Internet]. 2020 [cited 2022 Feb 14]. Available from: https://www.nicd.ac.za/first-case-of-covid-19-announced-an-update/

[40] National Institute for Communicable Diseases (NICD). Laboratory confirmed cases of COVID-19 in South Africa [homepage on the Internet]. 2022 [cited 2022 Feb 14]. Available from: https://www.nicd.ac.za/wp-content/uploads/2022/02/COVID-19-Weekly-Epidemiology-Brief-week-5-2022.pdf

[41] Koster W, Mutegi EM, Ocen F, Contexts for developing of national essential diagnostics list. Lessons from a mixed-methods study of existing documents, stakeholders and decision making on tier-specific essential in-vitro diagnostics in African countries. PLoS Global Public Health. 2023;3(5):e0001893. 10.1371/journal.pgph.0001893 37200237 PMC10194858

[42] World Health Organization (WHO). Selection of essential in vitro diagnostics at country level: Using the WHO model list of essential in vitro diagnostics to develop and update a national list of essential in vitro diagnostics [homepage on the Internet]. 2021 [cited 2025 Jan 24]. Available from: https://iris.who.int/bitstream/handle/10665/343385/9789240030923-eng.pdf?sequence=1

[43] Bonini P, Plebani M, Ceriotti F, Rubboli F. Errors in laboratory medicine. Clin Chem. 2002;48(5):691–698. 10.1093/clinchem/48.5.691 11978595

[44] Aarts J, Koppel R. Implementation of computerized physician order entry in seven countries. Health Aff. 2009;28(2):404–414. 10.1377/hlthaff.28.2.404 19275996

[45] Kuperman GJ, Gibson RF. Computer physician order entry: Benefits, costs, and issues. Ann Intern Med. 2003;139(1):31–39. 10.7326/0003-4819-139-1-200307010-00010 12834316

[46] Stevens WS, Cunningham B, Cassim N, Gous N, Scott LE. Cloud-based surveillance, connectivity, and distribution of the GeneXpert analyzers for diagnosis of tuberculosis (TB) and multiple-drug-resistant TB in South Africa. In: Persing DH, Tenover FC, Hayden RT, Ieven M, Miller MB, Nolte FS, Tang Y, Belkum A, editors Molecular microbiology: diagnostic principles and practice. New York, NY: Wiley, 2016; p.707–718.

